# Unraveling the metabolic effects of benzophenone-3 on the endosymbiotic dinoflagellate *Cladocopium goreaui*

**DOI:** 10.3389/fmicb.2022.1116975

**Published:** 2023-03-01

**Authors:** Kaidian Zhang, Zhen Shen, Weilu Yang, Jianing Guo, Zhicong Yan, Jiashun Li, Jiamin Lin, Xiaocong Cao, Jia Tang, Zhaoqun Liu, Zhi Zhou, Senjie Lin

**Affiliations:** ^1^State Key Laboratory of Marine Resource Utilization in South China Sea, Hainan University, Haikou, China; ^2^Key Laboratory of Tropical Hydrobiology and Biotechnology of Hainan, Hainan University, Haikou, China; ^3^State Key Laboratory of Marine Environmental Science, College of Ocean and Earth Sciences, Xiamen University, Xiamen, Fujian, China; ^4^Department of Marine Sciences, University of Connecticut, Groton, CT, United States

**Keywords:** oxybenzone, UV filters, toxic effects, *Cladocopium goreaui*, amino acid metabolism, coral reef ecosystems

## Abstract

As a well-known pseudo-persistent environmental pollutant, oxybenzone (BP-3) and its related organic ultraviolet (UV) filters have been verified to directly contribute to the increasing mortality rate of coral reefs. Previous studies have revealed the potential role of symbiotic Symbiodiniaceae in protecting corals from the toxic effects of UV filters. However, the detailed protection mechanism(s) have not been explained. Here, the impacts of BP-3 on the symbiotic Symbiodiniaceae *Cladocopium goreaui* were explored. *C. goreaui* cells exhibited distinct cell growth at different BP-3 doses, with increasing growth at the lower concentration (2 mg L^–1^) and rapid death at a higher concentration (20 mg L^–1^). Furthermore, *C. goreaui* cells showed a significant BP-3 uptake at the lower BP-3 concentration. BP-3 absorbing cells exhibited elevated photosynthetic efficiency, and decreased cellular carbon and nitrogen contents. Besides, the derivatives of BP-3 and aromatic amino acid metabolism highly responded to BP-3 absorption and biodegradation. Our physiological and metabolic results reveal that the symbiotic Symbiodiniaceae could resist the toxicity of a range of BP-3 through promoting cell division, photosynthesis, and reprogramming amino acid metabolism. This study provides novel insights into the influences of organic UV filters to coral reef ecosystems, which urgently needs increasing attention and management.

## Introduction

As a common ultraviolet absorbent, oxybenzone (benzophenone-3, hereafter BP-3) has been an ingredient in sunscreens and other personal care products including skin creams, cosmetics, shower gels, and shampoos, for over 40 years (Balmer et al., 2005; Mao et al., 2017). However, recently a mass of BP-3 residues have been detected in various environments including water bodies, sediment, and soil (Teoh et al., 2020). It has been estimated that thousands of tons of BP-3 are imported into the marine environment annually due to sewage discharge and recreational activities (e.g., swimming or diving) (Balmer et al., 2005; Goksøyr et al., 2009; Pintado-Herrera et al., 2017; Mitchelmore et al., 2019). Studies have identified that worldwide BP-3 concentrations in seawater range from nanogram to milligram per liter (Kim and Choi, 2014; Tsui et al., 2014; O’Donovan et al., 2020). Due to the increasing recreational tourisms in the reef zone, the coral reef ecosystem is undoubtedly a high-risk area for BP-3 pollution (Mitchelmore et al., 2021). However, BP-3 has clear toxicity to aquatic biota in both freshwater and other marine organisms (Paredes et al., 2014; Campos et al., 2017; Mao et al., 2017; Seoane et al., 2017; He et al., 2019; Lozano et al., 2020). Therefore, the potential threat of BP-3 to coral health has received wide attention worldwide (Moeller et al., 2021).

Coral reefs provide habitants of about one third of marine species and become one of the most biodiverse and economically valuable ecosystems on the planet (Plaisance et al., 2011; Pendleton et al., 2019). In past decades, global coral reefs experienced massive mortality due to an array of climate change impacts and anthropogenic-derived stressors, including sustained global warming, coastal eutrophication, overfishing, and chemical industrial pollution (Hoegh-Guldberg et al., 2007; Hughes et al., 2018; Sully et al., 2019). In particular, there have been growing concerns about the effects of UV screens on corals since 2008 (Danovaro et al., 2008). Downs et al. (2016) observed coral bleaching and death after acute BP-3 exposure (≤24 h) in *Stylophora pistillata*. So far, increasing laboratory studies have investigated the impact of BP-3 on the physiologies of corals, including photosynthetic yield, growth, bleaching, mortality, and the toxicity of BP-3 on larval and adult life stages of intact corals (Stien et al., 2018, 2020; Fel et al., 2019; He et al., 2019; Wijgerde et al., 2020). However, the toxicological mechanisms of BP-3 on corals have been contended. Recent researches implicated the metabolic products of BP-3, phototoxic oxybenzone-glucoside conjugates, can be considered as a culprit of the increasing bleaching rate of corals (Hansel, 2022; Vuckovic et al., 2022). On the contrary, it also has been revealed that Symbiodiniaceae could protect the coral host from the toxic effects of BP-3 metabolites by sequestering the phototoxins (Vuckovic et al., 2022). However, the protection mechanism by which Symbiodiniaceae protects the host remains unclear.

The endosymbiotic dinoflagellates Symbiodiniaceae are essential photosynthetic symbionts to the tropical and subtropical coral reef ecosystems. In oligotrophic tropical-subtropical shallow waters, algal symbionts absorb metabolic waste from the coral host and supply photosynthetically fixed carbon to coral hosts in return (Baker, 2003; Ceh et al., 2013). Until recently, seven Symbiodiniaceae lineages, from Clade A to G, are formally described according to systematic genetics and ecology analysis (LaJeunesse et al., 2018). Of these, *Cladocopium goreaui*, a type species of Symbiodiniaceae Clade C with physiological diversity, most species-specificity, and broad distribution, forms mutualistic associations with a broad diversity of hosts and is an important contributor to coral’s adaption in a wide range of irradiances and temperatures (LaJeunesse et al., 2018; González-Pech et al., 2019). Therefore, it is an excellent model for studying the responses of *C. goreaui* to UV screens and understanding symbiont’s protection mechanism against the toxicity of the UV screens in coral-Symbiodiniaceae holobionts.

In the present study, we investigated the metabolic and physiological responses of the symbiotic dinoflagellate *C. goreaui* to BP-3. Untargeted metabolomics in combination with physiological measurements were used to decipher the survival and molecular adaptive mechanisms of *C. goreaui* to BP-3 exposure. Our results provide a new perspective for furthering the understanding of detoxification mechanisms of coral-Symbiodiniaceae symbionts to environmental pollutants.

## Materials and methods

### Algal culture and experimental setup

The *C. goreaui* strain CCMP 2,466 was obtained from National Center for Marine Algae and Microbiota (NCMA). Prior to experiments, cells were cultured in L1 medium (Guillard, 1975) prepared with sterile-filtered (0.22 μm) seawater (30 PSU). Cultures were incubated at 25^°^C with a photon flux of 120 μE m^–2^ S^–1^ under a 12: 12 h light: dark cycle.

For the BP-3 treatments, the *C. goreaui* cells in the logarithmic growth phase were transferred into three experimental groups with different concentrations of BP-3 added: LBP-3 group (2 mg L^–1^ BP-3), HBP-3 group (20 mg L^–1^ BP-3), and control group (no BP-3), each in tuplicate and at a volume of 300 mL in conical flasks. The stock solution of BP-3 (CAS. No. 131-57-7) was prepared in methanol (HPLC grade) at the concentration of 5,000 mg L^–1^ and stored at –20^°^C in the dark. For LBP-3 and HBP-3 treatments, 0.12 and 1.20 mL of the stock was added to each of the 300 mL cultures, respectively.

### Cell growth and cell size measurement

The cell concentrations were determined daily using a Sedgewick–Rafter counting chamber (Phycotech, St. Joseph, MI, USA). Growth rate (μ) was calculated using μ = (ln N_1_–ln N_0_)/(t_1_–t_0_), where N_1_ and N_0_ represent the cell concentrations at t_1_ and t_0_, respectively (Zhang et al., 2021a). The average cell sizes were measured for each culture as the equivalent spherical diameter of over 500 cells using a Zeiss microscopy Axio Imager A2 (Carl Zeiss, Oberkochen, Germany) on the eighth day after incubation.

### Measurements of chlorophyll contents and photosynthetic rate

Chlorophyll contents were determined on the eighth day. Ten mL cultures of each group were collected by centrifugation at 5,000 *g*, 4^°^C for 10 min, and the cell pellets were immediately suspended in 4 mL pure acetone and kept in darkness at 4^°^C for 48 h to complete chlorophyll extraction. After centrifugation at 5,000 *g* for 10 min, the supernatants were separated to measure the chlorophyll contents using a UV-1100 Spectrophotometer (Mapada, Shanghai, China). The calculation of chlorophyll *a* (Chl *a*) and chlorophyll *c* (Chl *c*) contents was based on the equations from Jeffrey and Humphrey (1975) and Ritchie (2006). For the maximum photosystem II (PSII) quantum yield (*F*v/*F*m), two mL cultures were sampled daily, acclimated in darkness for 30 min, and used for the quantification of *F*v/*F*m using a Dual-PAM-100 Fluorometer (Walz, Effeltrich, Germany).

### Measurement of BP-3 concentration in the medium

To assess the potential of BP-3 degradation by abiotic factors (e.g., photolysis, volatilization) during the experiment, BP-3 added (2 mg L^–1^) cultures without algae were carried out and the BP-3 concentration in the medium was measured over time. Five mL cultures were collected at the start and end of the experiment, and centrifuged at 5,000 *g* for 10 min. Then the supernatant was subjected to the solid phase extraction (SPE) clean-up. Briefly, 20 ng isotopically-labeled standard was added in the supernatant and the mixture was passed through a hydrophilic-lipophilic balance (HLB) C18 SPE cartridge (200 mg, 6 mL) at a velocity of about 1 mL min^–1^. The cartridge was washed with 5 mL Milli-Q water after loading, followed by vacuum drying for 30 min. The target chemical was eluted with 5 mL of methanol and another 5 mL of methanol/acetone (1/1, v/v). The extract was concentrated under a gentle stream of high-purity nitrogen to less than 0.5 mL, and then reconstituted to 1 mL.

Subsequently, the concentration of BP-3 in each sample was quantified using high-performance liquid chromatography–tandem mass spectrometry (HPLC–MS/MS; TQ-S Micro, Waters, Milford, MA, USA) coupled with electro-spray ionization (ESI) tandem mass spectrometry (TQ-S Micro, Waters, Milford, MA, USA). The system was equipped with a reverse phase column (Acquity UPLC BEH C_18_, 50 mm length × 2.1 mm internal diameter; 1.7 μm particle size, Waters, Milford, MA, USA) connected with a guard column (Acquity UPLC BEH C_18_, 100 mm length × 2.1 mm internal diameter; 1.7 μm particle size, Waters, Milford, MA, USA), at a flow rate of 0.4 mL min^–1^. The mobile phases were 0.1% formic acid in Milli-Q water (mobile phase A) and acetonitrile/methanol (1/1, V/V) (mobile phase B). The gradient elution started with 10% B at 0 min, held for 1 min; linearly increased to 50% B at 1–2 min, then linearly increased to 75% B at 2–5 min, held for 5–10 min, then linearly increased to 100% B at 10–10.5 min, held for 10.5–14 min, and then the column was re-equilibrated to initial conditions at 14.1 min and stabilized for 2 min. The overall running time was 17 min. The injection volume was 2 μL and the column temperature was 40^°^C. Analytes were determined by ESI-MS/MS either in positive mode by multiple reaction monitoring (MRM). Turbo V ion source and MS/MS parameters were as follows: curtain gas (CUR), 10 psi; collision gas (CAD), medium; ion spray voltage, 4,000 V; temperature, 500^°^C; ion source gas 1 (GS1), 50 psi.

### Measurements of cellular carbon and nitrogen contents

About 2 × 10^6^ cells from each culture were filtered onto a pre-combusted (combusted in a Muffle Furnace at 450^°^C for 5 h) 25 mm GF/F membrane on the eighth day and stored at –80^°^C for subsequent elemental analysis. The cellular carbon (C) and nitrogen (N) contents were measured using a PE2400 SERIESII CHNS/O Elemental Analyzer (Perkin Elmer, Norwalk, CT, USA) as previously reported (Li et al., 2022). The weight of each element was divided by the cell number in the sample to obtain per cell content.

### Activity assay of T-AOC, GST, and caspase-3

Fifty mL cultures were harvested on the eighth day by centrifugation at 5,000 *g*, 4^°^C for 10 min, then the cell pellets were resuspended in 1 mL PBS (pH 7.4) and homogenized using a bead homogenizer (Bioprep-24, Allsheng Instruments Co. Ltd., China). After centrifugation at 14,000 *g*, 4^°^C for 10 min, the supernatant was used to detect various relevant activities. Total antioxidant capacity (T-AOC) and glutathione S-transferase (GST) detection kits (A015-1 and A004; Nanjing Jiancheng, China) were used for the measurements of T-AOC and GST activity, respectively. The caspase-3 activation level in different groups was determined with a Caspase-3 Colorimetric Assay Kit (KGA204; KeyGEN, China).

### Metabolite extraction and UPLC-MS analysis

About 1 × 10^7^ cells from each culture were collected on the eighth day by centrifugation at 5,000 *g*, 4^°^C for 10 min. The cell pellets were suspended with PBS (pH 7.4) and washed twice. Subsequently, metabolites in each sample were extracted. Firstly, two magnetic beads and 10 μL of the prepared internal standard (d3-Leucine, 13C9-Phenylalanine, d5-Tryptophan, and 13C3-Progesterone) were added to each sample. Then, 800 μL of precooled extraction reagent [methanol: acetonitrile: water (2:2:1, v/v/v)] was added into each sample and grind at 50 Hz for 5 min. After keeping at –20^°^C for 2 h, the ground samples were centrifuged at 25,000 *g*, 4^°^C for 15 min. Then 600 μL of each sample was transferred in split-new EP tubes and frozen dry. After that, 120 μL of 50% methanol was added to the dried sample and completely dissolved. After centrifugation at 25,000 *g*, 4^°^C for 15 min, the supernatant was used for further analysis.

The separation and detection of metabolites were analyzed using Waters UPLC I-Class Plus (Waters, Milford, MA, USA) tandom Q Exactive high resolution mass spectrometry (Thermo Fisher Scientific, USA). The chromatographic separation was performed on a Waters ACQUITY UPLC BEH C18 column (1.7 μm, 2.1 × 100 mm, Waters, Milford, MA, USA), and the column temperature was maintained at 45^°^C. The mobile phase consisted of 0.1% formic acid (A) and acetonitrile (B) in the positive mode. In the negative mode, the mobile phase consisted of 10 mM ammonium formate (A) and acetonitrile (B). The column was eluted with gradient conditions as follows: 0–1 min, 2% B; 1–9 min, 2–98% B; 9–12 min, 98% B; 12–12.1 min, 98% B–2% B; and 12.1–15min, 2% B (Flow rate 0.35 mL min^–1^, injection volume 5 μL).

The primary and secondary MS data acquisition were performed using Q Exactive (Thermo Fisher Scientific, Waltham, MA, USA). The full scan range was 70–1,050 m/z with a resolution of 70,000, and the automatic gain control (AGC) target for MS acquisitions was set to 3e6 with a maximum ion injection time of 100 ms. The top three precursors were selected for subsequent MS fragmentation with a maximum ion injection time of 50 ms and AGC of 1e5 under the resolution of 17,500. In this time, the stepped normalized collision energy was set as 20, 40, and 60 eV. ESI was set to the following parameters: flow rates of sheath gas and aux gas were 40 and 10, respectively; Spray voltage (| KV|) of positive-ion mode and negative-ion mode were 3.80 and 3.20, respectively; Capillary temperature and aux gas heater temperature was 320 and 350^°^C, respectively.

### Differential metabolites identification

The collected raw data were imported into a Compound Discoverer 3.3 software (Thermo Fisher Scientific, USA) to identify metabolites based on BMDB (BGI metabolome database), mzCloud database, and ChemSpider online database. The metaX software was used for further quality control (Wen et al., 2017). The screening of differentially changed metabolites between the control and LBP-3 groups [(LBP-3)/control comparison] was based on multivariate statistical analysis and univariate analysis. Firstly, the overall differences between the two groups were analyzed by principal component analysis (PCA) and partial least squares discriminant analysis (PLS-DA) (Barker and Rayens, 2003; Westerhuis et al., 2008). The variable importance in projection (VIP) value of metabolites in Orthogonal partial least squares discriminant analysis (OPLS-DA) (if OPLS-DA is over fitted, the VIP value of PLS-DA is used), fold change (FC), and q-value were used to filter differential metabolites. FC was obtained by FC analysis, *p*-value was obtained by *T*-test, and Q-value was obtained by Benjamini-Hochberg (BH) correction on *p*-value. In this study, the differential metabolites were identified according to the following criteria: (1) VIP of OPLS-DA model ≥ 1; (2) FC ≥ 1.2 or ≤ 0.83; (3) q-value < 0.05. And the metabolic pathway enrichment analysis was performed based on the KEGG database with a rigorous threshold (q-value < 0.05).

### Data analysis and statistical evaluation

All experiments were performed in tuplicate (*n* = 6), and the data were processed to obtain means with standard deviations (Mean ± SD). Statistical analyses were performed using the software SPSS (version 16.0; IBM, US). In order to evaluate the statistical significance of the differences between control and BP-3 groups, we performed the normal distribution test and homogeneity of variances test, and then one-way analysis of variance (ANOVA) was carried out to evaluate the significant differences in physiological parameters. Statistical significance (*) was determined at the level of *p* < 0.05.

## Results

### Algal growth under different BP-3 conditions

**FIGURE 1:**
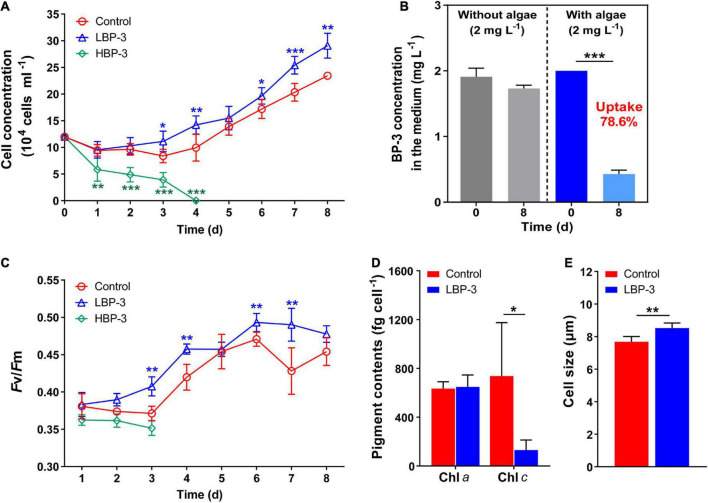
Physiological responses of *C. goreaui* to benzophenone-3 (BP-3). **(A)** Cell concentration. **(B)** Photodegradation of BP-3 under abiotic conditions (left) and BP-3 uptake by *C. goreaui* under the LBP-3 condition (right) over 8 days. **(C)** Photosystem II (PS II) maximum photochemical yield (*F*v/*F*m). **(D)** Pigment contents. **(E)** Cell size. Each data point is the mean from six replicates with the error bar indicating standard deviation (mean ± SD, *n* = 6). Asterisks (*p < 0.05; **p < 0.01; ***p < 0.001) indicate significant differences between different groups.

With the same initial cell concentration (1.1 × 10^5^ cells mL^–1^), the growth of *C. goreaui* under different BP-3 conditions started to diverge as soon as the first day (Figure 1A). A high concentration of BP-3 (20 mg L^–1^) addition strongly suppressed algal growth (Figure 1A), with the cell concentration declining rapidly and reaching zero on the fourth day (Figure 1A). Therefore, for the HBP-3 group, no more measurements were carried out after day four. In contrast, rapid cell growth occurred in the LBP-3 group (2 mg L^–1^) from the first day. Surprisingly, the LBP group exhibited even higher growth than the control group, with an average growth rate (from day 1 to day 8) of 0.16 ± 0.02 day^–1^, which was significantly higher than that in the control group (0.13 ± 0.02 day^–1^) (*T*-test, *p* < 0.05) (Supplementary Figure 1).

### Uptake of BP-3 by the *C. goreaui* cells

There was no significant difference in the BP-3 concentration in the medium without algae between day 1 and day 8 (Figure 1B), indicating the negligible degradation of BP-3 by abiotic factors during the experiment. In the LBP-3 group, the BP-3 concentration in the medium on the eighth day decreased from 2 to 0.43 ± 0.06 mg L^–1^ (Figure 1B). Therefore, 78.59% of added BP-3 in the medium was absorbed by *C. goreaui* cells after 8 days of cultivation (Figure 1B).

### Photosynthetic efficiency, chlorophyll contents, and cell size under BP-3 conditions

The *F*v/*F*m in the HBP-3 group was the lowest among the three groups of cultures. In contrast, the *Fv/Fm* of *C. goreaui* increased in the LBP-3 group and remained significantly higher than that in control from day 2 on (*T*-test, *p* < 0.05) (Figure 1C). Some fluctuations were observed in chlorophyll contents in the LBP-3 group compared to the control group (Figure 1D). Though no significant change was noted in the Chl *a* content after BP-3 addition (2 mg L^–1^), the Chl *c* content in the LBP-3 group was 82% lower than that in control (*T*-test, *p* < 0.05) (Figure 1D). In addition, the cell size in the LBP-3 group was 11% larger than that in control on the eighth day (*T*-test, *p* < 0.05) (Figure 1E).

### Decreased cellular carbon, nitrogen contents under the LBP-3 condition

**FIGURE 2:**
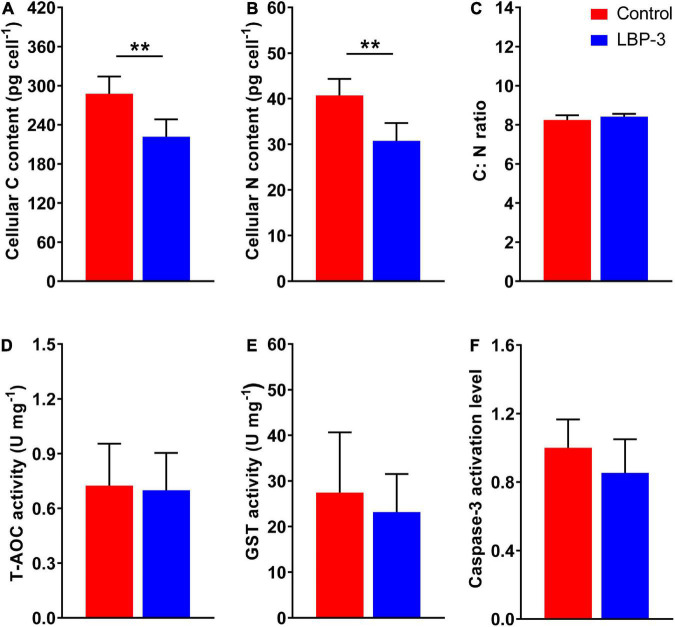
Cellular responses of *C. goreaui* in response to 2 mg L^– 1^ benzophenone-3 (BP-3) addition. **(A)** Cellular C content; **(B)** cellular N content; **(C)** C:N ratio; **(D)** total antioxidant capacity (T-AOC) activity; **(E)** glutathione S-transferase (GST) activity; **(F)** caspase-3 activation level. Data were collected from control and LBP-3 cultures (*n* = 6) after 8 days incubation. Each data point is the mean from six replicates with the error bar indicating standard deviation (mean ± SD, *n* = 6). **p < 0.01 indicate significant differences between different groups.

The cellular C content in the LBP-3 group decreased by 23% than that in the control group (*T*-test, *p* < 0.05) (Figure 2A). Similarly, cellular N content in the LBP-3 group was 25% lower than that in control (*T*-test, *p* < 0.05) (Figure 2B). Interestingly, the C: N ratio in *C. goreaui* cells seemed to be unaffected by BP-3 addition and remained relatively similar between the LBP-3 and control groups (Figure 2C).

### Response of T-AOC, GST activities, and caspase-3 level under the LBP-3 condition

Total antioxidant capacity and GST activities in *C. goreaui* cells were measured after BP-3 addition (2 mg L^–1^). Overall, no significant changes in T-AOC and GST activities were observed in the LBP-3 group compared with the control group (Figures 2D, E). The caspase-3 level in the LBP-3 group was 14.71% lower than that in the control group, but without statistical significance (Figure 2F).

### Metabolic alteration induced by BP-3

A total of 12 metabolome libraries were constructed from both the LBP-3 and control groups, and the metabolome of the LBP-3 group was compared with that of the control group. After data preprocessing and quality control, 11,047 metabolic compounds were identified in both positive and negative ion mode, and among them 2,874 was identified (Supplementary Table 1). The obvious separation of the metabolite profiles was observed by PCA analysis (Supplementary Figure 2). Furthermore, 318 upregulated and 396 downregulated differential metabolites were detected in the (LBP-3)/control comparison (Supplementary Figure 3 and Supplementary Table 2), revealing a dramatic response of *C. goreaui*’s metabolomic landscape to BP-3 addition.

### Functional distribution of the differentially expressed metabolites in the LBP-3 versus control comparison

**FIGURE 3:**
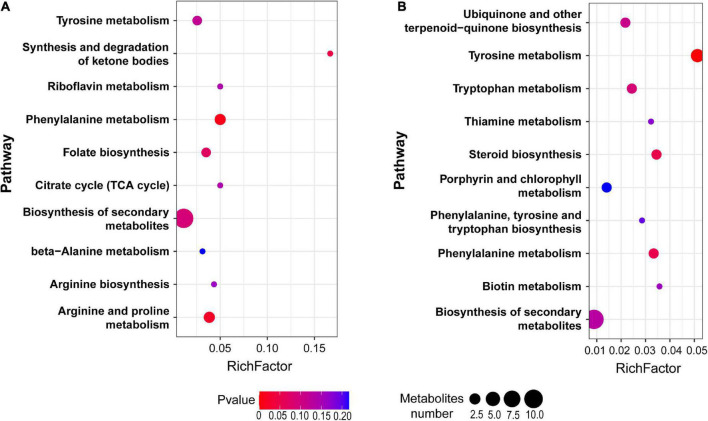
Pathway enrichment from the upregulated **(A)** and downregulated **(B)** metabolites between LBP-3 and control groups, respectively.

**FIGURE 4:**
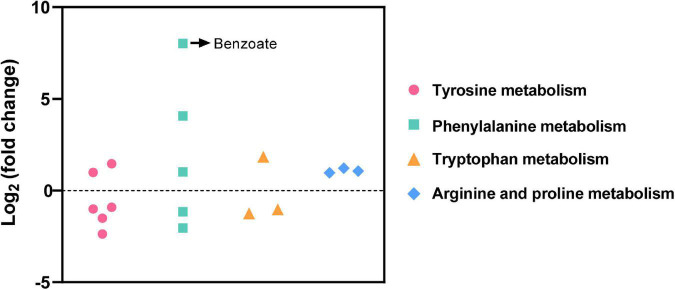
Differential metabolites related to amino acid metabolism in the (LBP-3)/control comparison.

Among the total 714 differential metabolites in the (LBP-3)/control comparison, the 318 upregulated metabolites were mainly related to amino acid (AA) metabolism, biosynthesis of secondary metabolites, synthesis and degradation of ketone bodies, and citrate cycle (Figure 3A and Supplementary Table 3). Similarly, the 396 downregulated metabolites were enriched to AA metabolism, ubiquinone and other terpenoid-quinone biosynthesis, biosynthesis of secondary metabolites, biotin metabolism, and porphyrin and chlorophyll metabolism (Figure 3B and Supplementary Table 4). For aromatic AA metabolism pathways, six metabolites in the tyrosine (Tyr) metabolism, five metabolites in the phenylalanine (Phe) metabolism (including a staggering 261-fold increase of benzoate), and three metabolites in the tryptophan (Trp) metabolism showed differential abundances in the (LBP-3)/control comparison (Figure 4). Another important AA metabolism affected by BP-3 addition was arginine (Arg) and proline (Pro) metabolism. Three metabolites in Arg and Pro metabolism, including L-arginine, 5-guanidino-2-oxopentanoic acid, and N2-succinyl-l-glutamic acid 5-semialdehyde, were significantly elevated in the LBP-3 group (Figure 4 and Supplementary Table 3).

### Differential concentration of benzene and substituted derivatives under the LBP-3 condition

To explore the potential biodegradation mode of BP-3 in *C. goreaui*, the significantly responded benzene and substituted derivatives were screened in the (LBP-3)/control comparison. Thirty-nine benzene and substituted derivatives were differentially changed, including 22 upregulated and 17 downregulated. The most dramatically changed were 2,3-dihydroxypropyl 3,4,5-trihydroxybenzoate, 3-phenoxybenzoic acid (3-PBA), benzoyl peroxide, and benzoate, which showed as high as 664-fold, 648-fold, 419-fold, and 261-fold increases in LBP-3 relative to control, respectively (Table 1).

**TABLE 1:** The information of benzene and substituted derivatives in the (LBP-3)/control comparison of *C. goreaui.*

Metabolite ID	Formula	Chemical structure	Name	MW^a^	FC^b^	q-value	VIP	Up/ Down
6.626_244.05836	C_10_H_12_O_7_	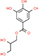	2,3-dihydroxypropyl 3,4,5-trihydroxybenzoate	244.06	664.44	6.14E-10	3.53	Up
7.185_214.06272	C_13_H_10_O_3_	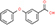	3-phenoxybenzoic acid	214.06	647.80	3.91E-08	3.52	Up
7.027_242.05802	C_14_H_10_O_4_	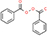	Benzoyl peroxide	242.06	418.53	6.19E-15	3.40	Up
6.046_104.02634	C_7_H_5_O_2_	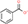	Benzoate	104.03	260.74	1.61E-08	3.27	Up
7.691_352.19167	C_22_H_26_NO_3_	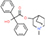	Clidinium	352.19	13.42	4.83E-06	2.23	Up
7.739_341.16268	C_20_H_23_NO_4_	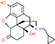	Naltrexone	341.16	11.98	3.08E-05	2.15	Up
4.102_290.13789	C_14_H_18_N_4_O_3_	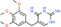	Trimethoprim	290.14	9.12	3.48E-04	2.01	Up
4.189_364.10906	C_17_H_20_N_2_O_5_S	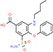	Bumetanide	364.11	6.79	4.38E-06	1.87	Up
6.567_420.14830	C_22_H_21_ClN_6_O	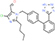	Losartan carboxaldehyde	420.15	4.28	7.20E-07	1.65	Up
4.317_122.08464	C_7_H_10_N_2_	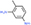	2,4-diaminotoluene	122.08	3.92	1.40E-03	1.59	Up
7.195_204.11535	C_13_H_16_O_2_	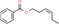	(z)-hex-3-enyl benzoate	204.12	3.75	4.47E-03	1.42	Up
4.032_293.16265	C_17_H_19_N_5_	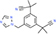	Anastrozole	293.16	3.49	8.02E-03	1.36	Up
3.78_256.12486	C_20_H_16_	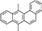	DMBA	256.12	2.46	3.40E-04	1.25	Up
4.532_215.09486	C_13_H_13_NO_2_	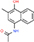	N-acetyl vitamin k5	215.09	2.44	8.13E-04	1.24	Up
4.236_180.04264	C_9_H_8_O_4_	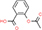	Aspirin	180.04	2.31	2.62E-05	1.26	Up
4.013_236.15255	C_13_H_20_N_2_O_2_	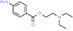	Procaine	236.15	2.21	1.94E-03	1.18	Up
3.65_270.10384	C_12_H_18_N_2_O_3_S	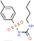	Tolbutamide	270.10	2.15	8.56E-03	1.14	Up
3.362_121.08943	C_8_H_11_N	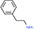	Phenethylamine	121.09	2.03	3.54E-03	1.06	Up
7.642_169.08946	C_12_H_11_N	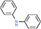	Diphenylamine	169.09	2.02	8.70E-03	1.02	Up
5.102_196.03700	C_9_H_8_O_5_	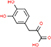	3,4-dihydroxyphenylpyruvic acid	196.04	1.99	1.35E-02	1.09	Up
0.567_255.00807	C_9_H_7_Cl_2_N_5_	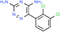	Lamotrigine	255.01	1.98	1.38E-04	1.11	Up
4.817_178.07450	C_9_H_10_N_2_O_2_	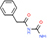	Phenacemide	178.07	1.86	9.00E-04	1.04	Up
3.258_233.98766	C_7_H_7_ClN_2_O_3_S	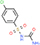	4-chlorobenzenesulfonylurea	233.99	0.28	3.30E-02	1.24	Down
7.986_179.13134	C_11_H_17_NO	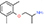	Mexiletine	179.13	0.31	9.92E-06	1.47	Down
3.977_222.08891	C_12_H_14_O_4_	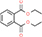	Diethyl phthalate	222.09	0.32	2.67E-05	1.45	Down
4.204_258.03184	C_11_H_12_Cl_2_N_2_O	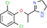	Lofexidine	258.03	0.34	7.58E-05	1.43	Down
4.073_223.04829	C_10_H_9_NO_5_	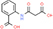	2-(carboxyacetamido)benzoic acid	223.05	0.35	1.97E-03	1.34	Down
7.156_165.11568	C_10_H_15_NO	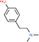	Hordenine	165.12	0.35	1.55E-04	1.36	Down
6.749_334.17766	C_19_H_26_O_5_	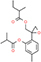	{2-[2-(isobutyryloxy)-4-methylphenyl]-2-oxiranyl}methyl 2-methylbutanoate	334.18	0.36	1.25E-03	1.30	Down
8.445_467.30392	C_29_H_41_NO_4_	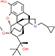	Buprenorphine	467.30	0.38	6.32E-05	1.33	Down
4.203_157.05308	C_10_H_7_NO	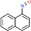	1-nitrosonaphthalene	157.05	0.42	5.09E-05	1.27	Down
4.423_180.09020	C_9_H_12_N_2_O_2_	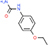	(4-ethoxyphenyl)urea	180.09	0.42	6.81E-04	1.20	Down
4.964_161.04797	C_9_H_9_NO_3_	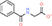	Hippurate	179.05	0.45	9.28E-04	1.17	Down
5.535_288.14739	C_16_H_20_N_2_O_3_	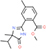	Imazamethabenz-methyl	288.15	0.47	1.62E-04	1.16	Down
9.927_426.24407	C_27_H_30_N_4_O	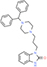	Oxatomide	426.24	0.48	4.71E-04	1.13	Down
1.516_136.06385	C_7_H_8_N_2_O	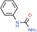	Yu0650000	136.06	0.51	4.67E-03	1.04	Down
9.453_308.21140	C_17_H_28_N_2_O_3_	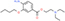	Oxybuprocaine	308.21	0.53	1.09E-03	1.04	Down
5.756_322.13170	C_19_H_18_N_2_O_3_	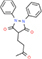	Kebuzone	322.13	0.53	6.10E-03	1.05	Down
1.29_185.06915	C_8_H_7_O_4_	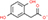	Homogentisate	185.07	0.53	1.38E-04	1.06	Down

^a^MW, molecular weight.

^b^FC, fold change.

## Discussion

It has been reported that the metabolites of BP-3 can increase the mortality rate of scleractinian corals, especially when the presence of BP-3 was combined with other stresses (Hansel, 2022). Meanwhile, symbiotic Symbiodiniaceae perform a potential role in the removal of BP-3 metabolites from the coral host (Vuckovic et al., 2022), but the protective mechanism has been poorly explored. In this study, using the *in vitro* batch culture of the type species of Symbiodiniaceae clade C, *C. goreaui*, we found that the exposure on low BP-3 resulted in increased photosynthetic efficiency, larger cell size, quicker growth rate, and decreased cellular C and N contents in *C. goreaui* cells. These responses manifested both at physiological and metabolomic levels. Our results demonstrated that *C. goreaui*, and possibly other symbiotic Symbiodiniaceae, are able to effectively metabolize BP-3 at low concentrations and gain growth advantages through a series of metabolic reprogramming.

### BP-3 uptake and biodegradation in *C. goreaui* cells

In this study, we monitored the BP-3 concentration in the medium of LBP-3 *C. goreaui* cultures and observed a significant decrease on BP-3 concentration after an 8 days incubation (Figure 1C). This result indicated that *C. goreaui* cells were able to take up BP-3 from the medium, which is in accordance with previous observations on other phytoplankton (Mao et al., 2017).

**FIGURE 5:**
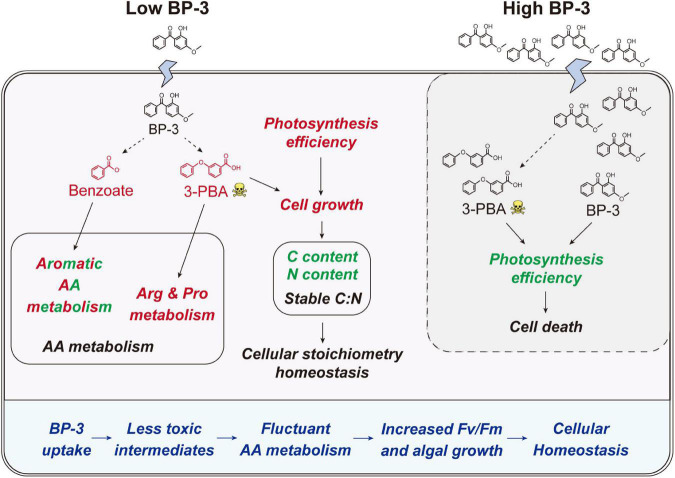
Schematic of the physiological and metabolic response in *C. goreaui* cells to BP-3 addition. Red and green fonts indicate upregulated and downregulated metabolites or related metabolic activities after BP-3 addition, respectively. Dotted arrows represent the potential metabolic pathways for BP-3. BP-3, oxybenzone; 3-PBA, 3-phenoxybenzoic acid; AA, amino acid.

After BP-3 absorption, benzene and substituted derivatives in *C. goreaui* showed significant increases (Table 1). Similarly, in the freshwater chlorophyte *Scenedesmus obliquus*, BP-3 could be degraded into benzene-containing intermediates, which are less toxic, after exposure to 3 mg L^–1^ BP-3 (Lee et al., 2020). In the BP-3-grown *C. goreaui*, 3-PBA, an important intermediate metabolite of pyrethroid (Zhao et al., 2020), showed an astounding 648-fold increase (Table 1). Due to a high solubility, strong mobility, and long half-life (Topp and Akhtar, 1990; Yuan et al., 2010), 3-PBA is able to harm the reproductive function, immune system, and endocrine system of animals (Sun et al., 2007; Han et al., 2008; Zhang et al., 2010). The high rate of mortality in *C. goreaui* with high BP-3 exposure probably mirrors the negative effects of this degradation product (Figure 5). In addition, as the key intermediate for Phe metabolism (Wildermuth, 2006; Gonda et al., 2018), benzoate also showed a staggering 261-fold increase in the LBP-3 group (Figure 4 and Supplementary Table 2). These results indicate that the biodegradation of BP-3 by *C. goreaui* cells could remodel the benzene-containing secondary metabolites, thereby affecting cellular metabolic regulation (Figure 5).

### BP-3 could affect symbiodiniaceae growth

At low BP-3 exposure level (2 mg L^–1^), *C. goreaui* cells exhibited increased growth (Figure 1A), agreeing with recent observations on the freshwater chlorophyte *Scenedesmus. quadricauda* (Teoh et al., 2020). Wijgerde et al. (2020) also found that the Symbiodiniaceae density in the coral *Stylophora pistillata* increased slightly (although not statistically significant) when exposed to BP-3. Recently, increasing studies demonstrated that the intracellular contaminants could be diluted by quick cell division, and phytoplankton can reduce the negative effects of toxic substance through faster proliferation (Skoglund and Swackhamer, 1994; Tikoo et al., 1997; Mao et al., 2017). Hence, the elevated growth of BP-3-grown *C. goreaui* might be a mitigation mechanism for toxicity mitigation. Meanwhile, the cell size of *C. goreaui* increased when exposed to 2 mg⋅L^–1^ BP-3 (Figure 1F). Changes in cell size is a typical stress response (particularly nutrient deficiencies) in phytoplankton due to the inhibition of cell division (Li et al., 2016; Teoh et al., 2020; Wang et al., 2022). However, the LBP-3 treated cultures actually exhibited a higher cell division rate than the control. The cell size enlargement might result from a different reason. For instance, bigger cells can lead to slower uptake and alleviative propensity to biotoxicity (Duan et al., 2017), potentially another toxicity mitigation mechanism.

Benzophenone-3 exposure also induced a significant decrease in the Chl *c* content (Figure 1D). Correspondingly, there was a downregulation in porphyrin and chlorophyll metabolism in *C. goreaui*, as shown in the metabolomic data (Figure 3B). This result indicates that BP-3 may disrupt the pigment synthesis in *C. goreaui* cells. Interestingly, increased photosynthetic efficiency was also observed in *C. goreaui* growth in the LBP-3 cultures (Figure 1C). The disparate effects of BP-3 on Chl *a*, photosynthetic efficiency, and growth rate versus Chl *c* is quite strikingly and might reflect the fact that Chl *a* is the major pigment that executes photosynthesis (Suggett et al., 2003). The enhanced photosynthetic efficiency in BP-3-grown *C. goreaui* cells can potentially meet the elevated energy demand imposed for the biodegradation of BP-3 and its derivatives, and the faster cell division for the toxicity dilution.

After 2 mg L^–1^ BP-3 exposure, *C. goreaui* showed no significant changes in T-AOC and GST activities (Figures 2D, E), indicating that cells did not experience significant toxic (oxidative stress) effects at this dosage. With all results taken together, it is rather clear that *C. goreaui* can carry out a series of toxicity mitigation strategies, including rapid growth, expanded cell size, and elevated photosynthesis, to minimize the cytotoxicity of low concentration BP-3. However, based on the lethal effects of the high concentration we used (20 mg L^–1^), there seems to be a threshold concentration, beyond which BP-3 could damage the photosynthesis system and cause rapid death of *C. goreaui* cells (Figure 5).

### Effects of BP-3 on cellular nutrition regulation

Besides algal growth, BP-3 exposure also highly impacted the cellular metabolisms in *C. goreaui*. In marine bacterium *Epibacterium mobile*, BP-3 exposure has been found to result in perturbation of AA metabolism (Lozano et al., 2021). Similarly, significant changes in metabolites were noted in our present study, which impacted major AA metabolism pathways in BP-3 exposed *C. goreaui* (Figure 4). The BP-3 biodegradation in microalgae can produce a series of aromatic compounds, which are important precursor species for AA synthesis, especially Phe, Tyr, and Trp (Pérez et al., 2002; Gosset, 2009). Here, these three aromatic AA metabolites were all significantly affected in BP-3 exposed *C. goreaui* cells (Figure 4), implying the potential interference of intracellular BP-3 metabolites with AA metabolism (Figure 5). In addition, Arg and Pro metabolism was significantly enriched by upregulated metabolites in BP-3 absorbed *C. goreaui* cells (Figure 4). Arg and Pro are the proteinogenic AA which are essential for cellular primary metabolism (Szabados and Savouré, 2010; Trovato et al., 2019). Increasing Arg and Pro accumulation could confer biotic and abiotic stress tolerance, such as UV irradiation (Park et al., 2020), heavy metals (Seneviratne et al., 2019), and oxidative stress (Momose et al., 2010; Nishimura et al., 2010; Forlani et al., 2019). Therefore, at low BP-3 exposure, *C. goreaui* cells may also resist the BP-3 stress *via* modulating Arg and Pro metabolism (Figure 5).

Besides AA metabolism, cellular element in *C. goreaui* was also affected by BP-3 exposure. The cellular N and C content in *C. goreaui* were significantly decreased under the LBP-3 condition (Figures 2A, B), indicating that cellular regulation of AA metabolism at low BP-3 exposure also affects N and C metabolism. As photosynthetic efficiency was elevated in cells exposed to a low dose of LBP-3 (Figure 1C), the depressed cellular C content might be due to a greater increase in cell division rate than in photosynthesis or that the apparently increased ATP production might to be used for the BP-3 biodegradation than to fuel C fixation. Meanwhile, we also noticed that *C. goreaui* cells in the LBP-3 cultures showed significantly decreased stearate content (Supplementary Table 3). Stearic acid is a long-chain saturated fatty acid functions as a crucial storage form of cellular C (Du et al., 2016). This might be another reason for the reduction in C content in LBP-3-grown cultures. Given the changes in C and N induced by BP-3 exposure, it is interesting to note that the C:N ratio of *C. goreaui* cells exhibited no statistical changes (Figure 2C). As we all know, elemental stoichiometry is crucial to map the cellular nutrient status in phytoplankton (Zhang et al., 2021b; Li et al., 2022). Our results indicate that the low BP-3 dose used in our study did not disrupt cellular stoichiometric homeostasis in *C. goreaui* cells (Figure 5).

## Conclusion

In coral reef ecosystems, endosymbiotic dinoflagellates are believed to be responsible for the detoxification of BP-3 and related UV filters in their coral hosts. In this study, we investigated the physiological and metabolic responses of symbiotic Symbiodiniaceae *C. goreaui* after BP-3 exposure. Overall, *C. goreaui* cells exhibited increased algal growth, elevated photosynthetic efficiency, decreased cellular C and N contents, and remodeled AA metabolism, potentially as multi-faceted means to cope with the toxic effects of absorbed BP-3. These findings shed light on how Symbiodiniaceae respond to BP-3 stress at low doses. However, the rapid algal death at high BP-3 concentration calls for further exploration to pinpoint the BP-3 tolerance threshold of Symbiodiniaceae. Information on the threshold will be crucial for assessing the environmental risk of organic UV filters to coral reef ecosystems and informing the formulation of coral reef reserve management regulation.

## Data availability statement

The data presented in this study are deposited in the MetaboLights repository, accession number MTBLS6890.

## Author contributions

KZ and ZZ conceived and designed the study. KZ, ZS, WY, JG, ZY, JLi, JT, and ZL performed the experiments, with the help of JLin and XC for BP-3 measurement. KZ, SL, and ZZ interpreted the data and wrote the manuscript. All authors read and approved the final version of the manuscript.
