# Supplementary material for: Unraveling the metabolic effects of benzophenone-3 on the endosymbiotic dinoflagellate Cladocopium goreaui
Source: Front Microbiol. 2023 Mar 1;13:1116975. doi: 10.3389/fmicb.2022.1116975 (PMC10016356; doi:10.3389/fmicb.2022.1116975)
Supplement: Supplementary file 1 [file Data_Sheet_1.PDF]

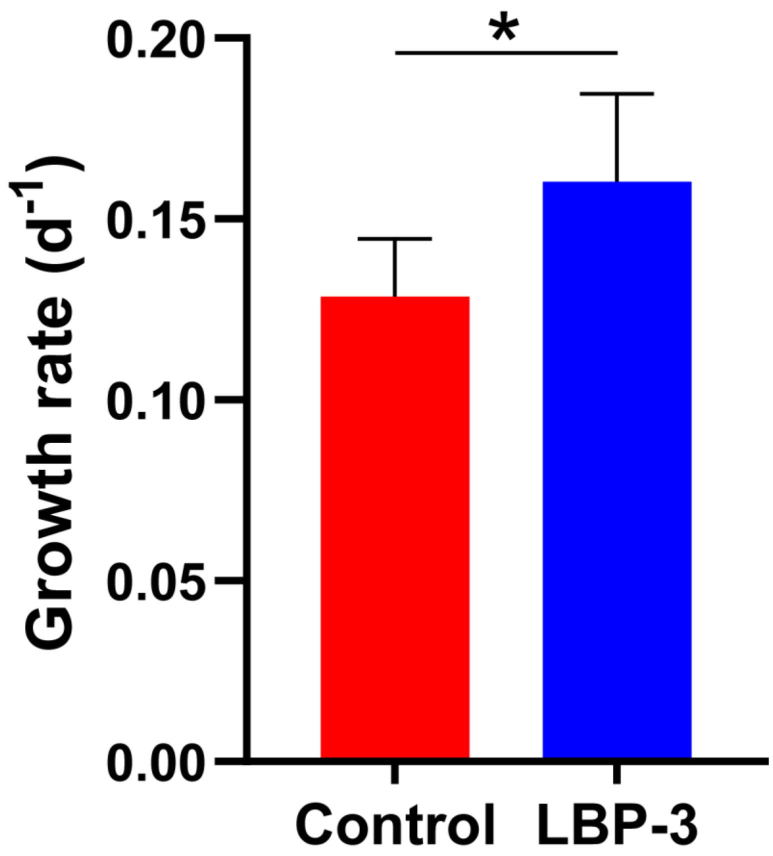

Figure S1. Average growth rate of the LBP-3 and control groups.

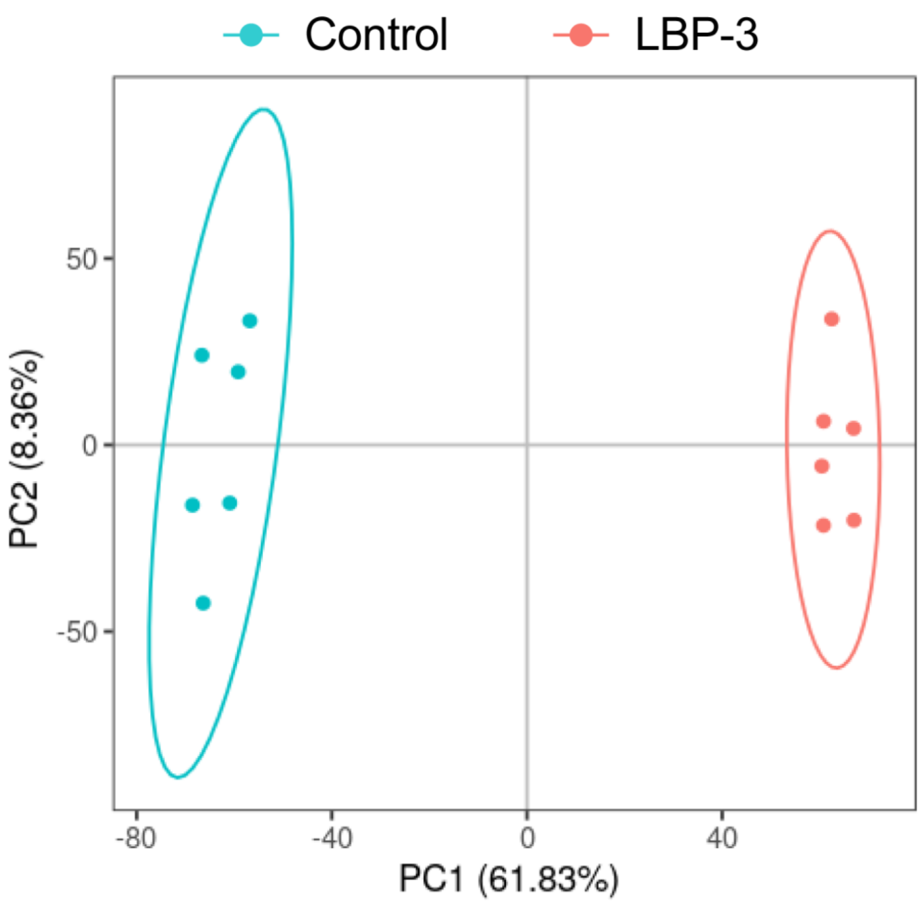

Figure S2. PCA score chart of the LBP-3 and control groups.

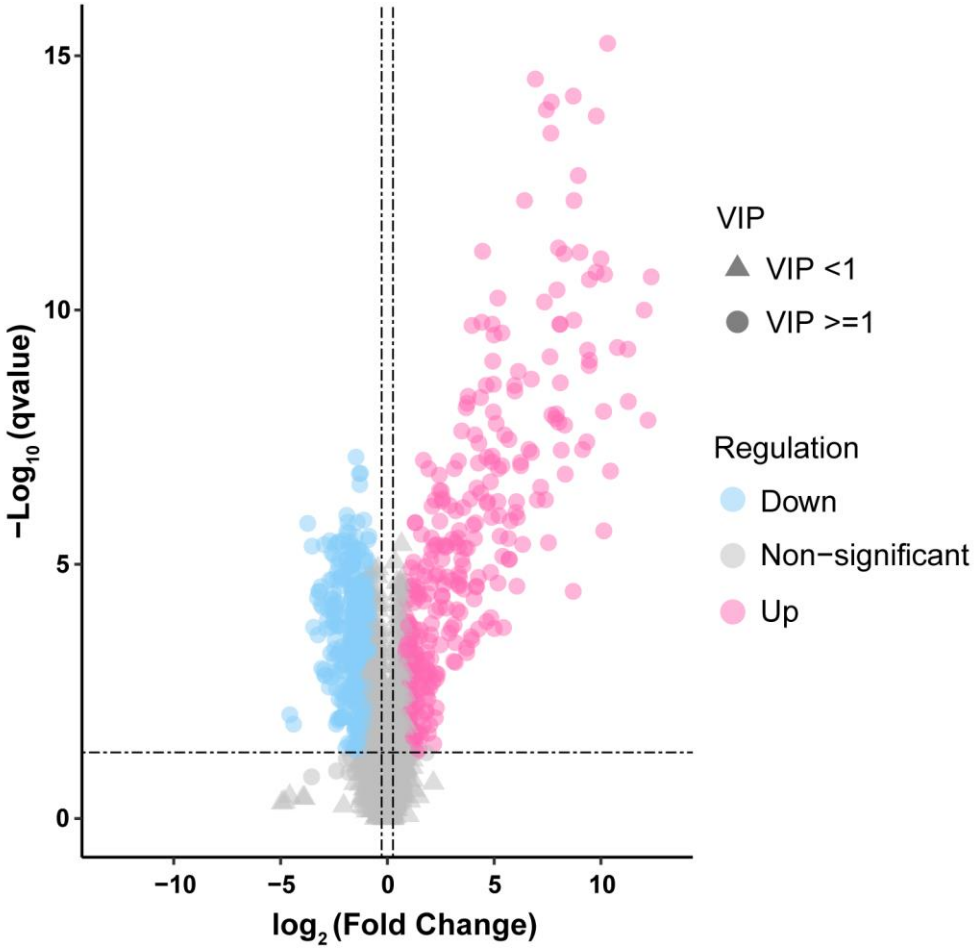

Figure S3. Volcano plot of differential metabolites in the (LBP-3)/Control comparison. The up-regulated and down-regulated significantly differential metabolites (VIP ≥ 1, Fold Change ≥ 1.2 or ≤ 0.83, q-value < 0.05) are labeled in pink and blue, respectively.
